# Results of Laparoscopic Surgery and D3 Lymph Node Dissection Combined With Chemotherapy for the Radical Treatment of Advanced-Stage Right Colon Cancer: A Single-Center Observational Study in Vietnam

**DOI:** 10.7759/cureus.43243

**Published:** 2023-08-09

**Authors:** Long Huynh Thanh, Khiem Nguyen Manh, Minh Nguyen Thi, Anh Nguyen Tri Trung, Kien Nguyen Trung, Thang Le Viet, Nung Vu Huy

**Affiliations:** 1 General Surgery, Nguyen Tri Phuong Hospital, Ho Chi Minh, VNM; 2 Oncology, Cancer Institute, 108 Military Central Hospital, Hanoi, VNM; 3 Oncology, Cancer Department, 198 Hospital, Hanoi, VNM; 4 Hematology and Blood Transfusion, Military Hospital 103, Hanoi, VNM; 5 Hematology and Blood Transfusion, Vietnam Military Medical University, Hanoi, VNM; 6 Nephrology and Hemodialysis, Military Hospital 103, Hanoi, VNM; 7 Nephrology and Hemodialysis, Vietnam Military Medical University, Hanoi, VNM; 8 Surgery, Vietnam Military Medical University, Hanoi, VNM

**Keywords:** right colon cancer, recurrence, mortality, laparoscopic complete mesocolic excision surgery, adjuvant chemotherapy

## Abstract

Aim: To describe the results of laparoscopic surgery and D3 lymph node dissection combined with adjuvant chemotherapy (ACT) for the treatment of advanced-stage right colon cancer (stages II and III).

Methods: A total of 172 right colon cancer patients (with tumour, node, and metastasis (TNM) stage II and III; mean age of 59.30±14.27 years; 58.1% male, 41.9% female) who had undergone complete mesocolic excision (CME) with D3 lymph node dissection at Nguyen Tri Phuong Hospital, Ho Chi Minh City, Vietnam, were included in this study. They were divided into two groups: group 1 (n=34) without ACT and group 2 (n=138) with ACT. We collected clinical and laboratory data twice (before and after one year of performing laparoscopic surgery). Rates of recurrence and mortality were obtained during a five-year follow-up.

Results: After one year of surgery, the rate of anemia and the increase in serum carcinoembryonic antigen (CEA) levels in group 1 were significantly higher than those in group 2 (p<0.001). After five years of follow-up, the recurrence rate was 11.6% (that of group 1 was 41.2%, which is higher than that of group 2, i.e., 4.3%; p<0.001), and the mortality rate was 8.7% (that of group 1 was 32.4%, which is higher than that of group 2, i.e., 2.9%; p<0.001). Preoperative serum CEA levels were predictive of recurrence and mortality, with an area under the curve (AUC) of 0.729 and 0.805, respectively (p<0.001).

Conclusions: Laparoscopic CME surgery and D3 lymph node dissection combined with ACT reduced the five-year recurrence and mortality rates for advanced-stage right colon cancer patients.

## Introduction

Colon cancer is a common malignancy, ranking second after gastric cancer among gastrointestinal cancers [1,2]. The current treatment for colorectal cancer is multimodal. In particular, radical surgery is considered the most effective treatment method [1-4]. Laparoscopic colorectal surgery has become a treatment method for many patients diagnosed with right colon cancer in particular and colorectal cancer in general [5-7]. Utilizing this technique offers benefits, such as decreased surgical invasiveness, decreased occurrences of postoperative complications, and quicker recuperation after surgery [8-10]. Complete mesocolic excision (CME) with D3 lymph node dissection is indicated and applied in many countries around the world in the surgery for right colon cancer [11-14].

 Despite radical surgery and D3 lymph node dissection treatment in patients with colon cancer, the recurrence rate after surgery is still about 29% in patients with stage II cancer and 42% in stage III cancer [15-18]. After radical surgery or in cases where there is a high risk of recurrence, adjuvant chemotherapy (ACT) is mandatory for patients with stage III cancer, whereas stage II cancer patients undergo ACT if the tumor has invaded the muscle layer or has invaded nerves and/or blood vessels. The prime objective of administering ACT is to eradicate the possibility of any residual microscopic disease, thus diminishing the chances of recurrence. According to research, on stage III colon cancer, ACT reduces the risk of recurrence by approximately 14% [18]. Although the advantage of ACT for treating patients with colon cancer is well-established and widely accepted, the initiation rate of ACT completion for patients with stage III colon cancer has been reported to be only 78% [19]. In Vietnam, colon cancer is often diagnosed late because patients come to the clinic late, so the cancer is already often at the invasive stage, with complications or distant metastases.

In this paper, we describe the results of laparoscopic surgery and D3 lymph node dissection combined with chemotherapy for the radical treatment of right colon cancer during five-year follow-up.

## Materials and methods

Study design

A descriptive, longitudinal, non-interventional observational study on 172 right colon cancer patients who had undergone laparoscopic CME with D3 lymph node dissection from November 2011 to November 2015 at Nguyen Tri Phuong Hospital, Ho Chi Minh City, Vietnam, were enrolled in our study. This study was approved by the Ethical Committees of Vietnam Military Medical University (approval no. 73/QĐ-HVQY) and Nguyen Tri Phuong Hospital (approval no. 112/QĐ-BVNTP). Based on the status of whether or not to use ACT after surgery, 172 patients were divided into two groups: group 1, which consisted of patients of laparoscopic CME with D3 lymph node dissection non-treated with ACT (n=34), and group 2, which consisted of patients of laparoscopic CME with D3 lymph node dissection treated with ACT (n=138). The study compared various factors, including demographics, operational information, pathologic results, short-term clinical outcomes after surgery, recurrence rates, and overall survival between the two groups. Patients with R1 and R2 resection or multiple synchronous cancers, combined organ resection, and stage IV colon cancer were excluded from this study. All patients were collected data on clinical characteristics and laboratory parameters at the time of surgery (T0).

Surgical procedure and D3 lymph node dissection

The surgical procedure was performed by a surgeon with more than 20 years of professional practice. The vascular pedicles were divided at their origin in right colon tumors, and the draining lymph nodes along the superior mesenteric vein were removed. D3 lymph node dissection was defined as the removal of primary lymph nodes at the root of the feeding vessels (ileocolic vessels and the right branch of the middle colic artery or middle colic artery), followed by vessel ligation at the origin site. All 172 patients were clinically examined, underwent laboratory tests, and had their cancer stage determined to indicate CME before surgery. Tumor characteristics, including size, differentiation, lymph node metastasis, and number and size of lymph nodes, were also collected after surgery.

Postoperative complications

Significant complications, such as severe bleeding requiring transfusions and organ damage requiring surgical interventions, were observed during the surgery. Following the surgery, various complications were examined using the Clavien-Dindo Classification, including infection at the site of surgery, blockage of the intestines, improper healing at the surgical site, bleeding, failure of organs, and sepsis. Surgical outcomes and early complications were collected from the end of surgery until the patient was discharged from the hospital.

ACT

In this study, all patients had indications to use ACT according to the FOLFOX (folinic acid, fluorouracil, and oxaliplatin) protocol, with 12 cycles, two weeks each, after surgery according to the instructions of the National Comprehensive Cancer Network (NCNN) and Vietnam's Ministry of Health. All 172 patients were explained of the benefits and risks of treatment, but only 138 agreed and used the full regimen. The remaining 34 patients refused to use this treatment protocol. During the time of taking the drug, we monitored the side effects of the drug, such as nausea, vomiting, and leukopenia.

Based on whether or not to use ACT after surgery, the patients were divided into two groups: group 1, which consisted of patients of laparoscopic CME with D3 lymph node dissection non-treated with ACT (n=34), and group 2, which included patients of laparoscopic CME with D3 lymph node dissection treated with ACT (n=138).

Follow-up

All 172 patients were followed up every three months for the first year after surgery and every six months for the next four years (five years in total). They all underwent colonoscopy once a year, and whole-body computed tomography (CT) were performed every six months in the first two years to monitor recurrence. Abdominal ultrasound and chest X-ray may be substituted from year three onwards.

Recurrence of the tumor was defined as in-situ or metastases in the liver, bone, peritoneum, lung, or brain. Any recurrence was identified using clinical or radiographic methods, and details, such as the date and type of recurrence, the reason and date of death, and any other contributing factors, were collected and recorded in the database. Survival time was calculated from the date of surgery to the date of death.

Body mass index (BMI), anemia, serum albumin, high-sensitivity C-reactive protein (hs-CRP), and CEA levels for the second time (T1) were determined at the end of the first year after surgery. Recurrence, metastasis, and mortality were collected during the five-year follow-up.

Statistical methods

The continuous normal distribution data were examined using the mean and standard deviation and analyzed through analysis of variance (ANOVA) and Student's t-test. The skewed distribution data were represented by the median (25th-75th percentiles) and analyzed by the Mann-Whitney U and Kruskal-Wallis tests. Categorical data were presented through frequency with percentages and analyzed using a chi-square test. Receiver operating characteristic (ROC) curves were used to determine the predicting value of some indices for mortality after five years of follow-up. Multivariable adjusted regression analysis was carried out to identify predictors of hospital mortality. Kaplan-Meier analysis was used to assess survival curves and evaluated with the log-rank test. IBM SPSS Statistics for Windows, Version 20 (released 2011; IBM Corp., Armonk, New York, United States) was utilized to perform statistical analysis, and a p-value less than 0.05 was considered significant.

## Results

The results in Table 1 show no significant differences in the demographic and laboratory characteristics between group 1 and group 2, except that the proportion of patients with serum albumin concentration <35.0 g/l was lower in group 1 than in group 2 with a p-value=0.028. 

**Table 1:** Comparison of the demographic and laboratory characteristics in group 1 and group 2 at the time of surgery (T0) BMI: body mass index; hs-CRP: high-sensitivity C-reactive protein; CEA: carcinoma embryonic antigen; TNM: tumor node metastasis; N/A: not applicable

Clinical characteristics and laboratory parameters	Total (n=172)	Group 1 (n=34)	Group 2 (n=138)	p-value
Age (years)	59.30 ± 14.27	61.79 ± 8.94	58.69 ± 15.27	0.126
Number of male (n, %)	100 (58.1)	17 (50)	83 (60.1)	0.283
Having combined disease (n, %)	84 (48.8)	13 (38.2)	71 (51.4)	0.167
Mean BMI (kg/m^2^)	21.33 ± 2.76	21.37 ± 2.67	21.32 ± 2.79	0.925
BMI < 18.5 kg/m^2^ (n,%)	29 (16.9)	7 (20.6)	22 (15.9)	0.231
BMI: 18.5-22.9 kg/m^2^ (n,%)	98 (57.0)	15 (44.1)	83 (60.1)	
BMI≥23.0 kg/m^2^ (n,%)	45 (26.2)	12 (35.3)	33 (23.9)	
Anemia (n,%)	56 (32.6)	9 (26.5)	47 (34.1)	0.398
Hemoglobin (g/L)	131.94 ± 15.08	132.44 ± 16.84	131.82 ± 14.68	0.832
Mean albumin (g/L)	38.01 ± 4.97	39.38 ± 4.59	37.67 ± 5.02	0.073
Albumin<35.0 g/L (n,%)	46 (26.7)	4 (11.8)	42 (30.4)	0.028
Median hs-CRP (mg/L)	3.5 (2.6-5.3)	3.2 (2.32-5.6)	3.5 (2.7-5.3)	0.519
hs-CRP>5.0 mg/L (n,%)	51 (29.7)	11 (32.4)	40 (29.0)	0.70
Macroscopic picture of wart form (n, %)	32 (18.6)	3 (8.8)	29 (21.0)	0.356
Macroscopic picture of ulcerative form (n, %)	12 (7.0)	1 (2.9)	11 (8.0)	
Macroscopic picture of ring form (n, %)	28 (16.3)	6 (17.6)	22 (15.9)	
Macroscopic picture of polyp form (n, %)	82 (47.7)	20 (58.8)	62 (44.9)	
Macroscopic picture of others (n, %)	18 (10.5)	4 (11.8)	14 (10.1)	
Median CEA (ng/mL)	6.07 (2.4-12.97)	6.3 (1.93-10.53)	6.0 (2.7-14.36)	0.632
CEA>5.0 ng/mL (n,%)	96 (55.8)	19 (55.9)	77 (55.8)	0.993
Median tumor size after surgery (cm)	3 (2-5)	3 (2-5)	3 (2-5)	0.966
Tumor size after surgery <3.0 cm (n, %)	51 (29.7)	10 (29.4)	41 (29.7)	0.970
Tumor size after surgery: 3.0-7.0 cm (n, %)	104 (60.5)	21 (61.8)	83 (60.1)	
Tumor size after surgery ≥7.0 cm (n, %)	17 (9.9)	3 (8.8)	14 (10.1)	
Highly differentiated tumor (n, %).	14 (8.1)	4 (11.8)	10 (7.2)	0.688
Moderately differentiated tumor (n, %).	131 (76.2)	25 (73.5)	106 (76.8)	
Poorly differentiated tumor (n, %)	27 (15.7)	5 (14.7)	22 (15.9)	
Lymph node metastasis (n, %)	85 (49.4)	18 (52.9)	67 (48.6)	0.646
Distant metastasis (n, %)	0 (0)	0 (0)	0 (0)	N/A
Nodule size <5 mm (n, %)	172 (100)	34 (100)	138 (100)	N/A
Nodule size 5-10 mm (n, %)	0 (0)	0 (0)	0 (0)	
Nodule size >10 mm (n, %)	0 (0)	0 (0)	0 (0)	
TNM stage II (n, %)	87 (50.6)	16 (47.1)	71 (51.4)	0.215
TNM stage III (n, %)	85 (49.4)	18 (52.9)	67 (48.6)	
Mean lympho node dissection during colectomy	17.53 ± 4.14	17.12 ± 3.50	17.64 ± 4.29	0.514
Median lymph node metastasis (median)	0 (0-2)	1 (0-2)	0 (0-1.25)	0.502

The results in Table 2 show that one year after surgery, group 1 had a higher rate of anemia and elevated CEA, a higher concentration of CEA, and a lower concentration of hemoglobin than those of group 2 (p<0.001). During five years of follow-up, the recurrence, metastasis, and death rates in group 1 were higher than those in group 2 (p<0.001). 

**Table 2:** Comparison of the demographic and laboratory characteristics after one year surgery and ratio of recurrence, metastasis, and mortality during five years of follow-up in group 1 and group 2 BMI: body mass index; hs-CRP: high-sensitivity C-reactive protein; CEA: carcinoma embryonic antigen

Clinical characteristics and laboratory parameters	Total (n=172)	Group 1 (n=34)	Group 2 (n=138)	p-value
Mean BMI (kg/m^2^)	20.73 ± 2.87	20.96 ± 2.90	20.67 ± 2.86	0.590
BMI <18.5 kg/m^2^ (n, %)	45 (26.2)	10 (29.4)	35 (25.4)	0.081
BMI: 18.5-22.9 kg/m^2^ (n, %)	92 (53.5)	13 (38.2)	79 (57.2)	
BMI ≥23.0 kg/m^2^ (n, %)	35 (20.3)	11 (32.4)	24 (17.4)	
Anemia (n, %)	73 (42.4)	26 (76.5)	47 (34.1)	< 0.001
Hemoglobin (g/L)	128.78 ± 14.98	116.44 ± 8.52	131.82 ± 14.68	< 0.001
Mean albumin (g/L)	36.77 ± 6.00	38.15 ± 6.41	36.43 ± 5.88	0.173
Albumin <35.0 g/L (n, %)	58 (33.7)	6 (17.6)	52 (37.7)	0.027
Median hs-CRP (mg/L)	2.1 (1.2-3.05)	2.1 (1.2-2.8)	2.1 (1.2-3.2)	0.861
hs-CRP >5.0 mg/L	26 (15.1)	5 (14.7)	21 (15.2)	0.941
Median CEA (ng/mL)	4.54 (3.07-31.93)	34.17 (31.65-45.83)	3.84 (2.7-6.08)	<0.001
CEA >5.0 ng/mL (n, %)	80 (46.5)	34 (100)	46 (33.3)	<0.001
Recurrence of cancer at the anastomosis (n, %)	20 (11.6)	14 (41.2)	6 (4.3)	<0.001
Metastasis (n, %)	18 (10.5)	13 (38.2)	5 (3.6)	<0.001
Mortality (n, %)	15 (8.7)	11 (32.4)	4 (2.9)	<0.001

**Figure 1:**
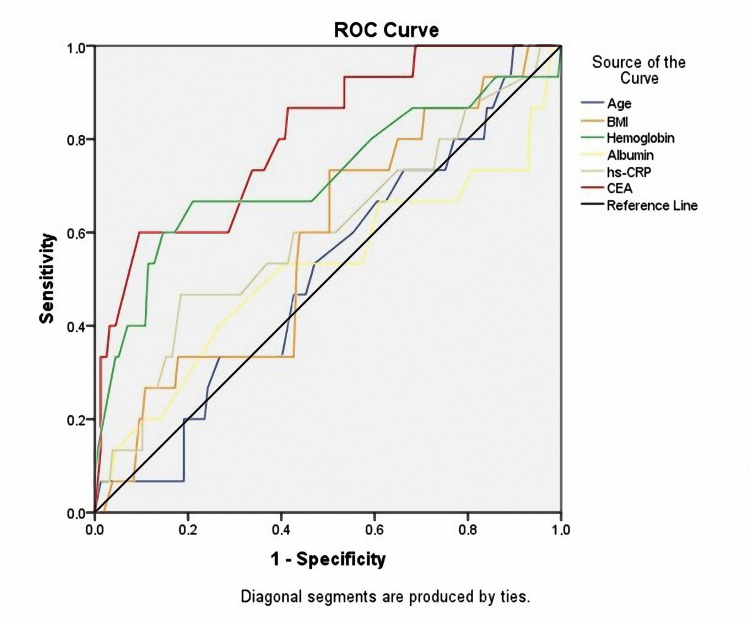
Receiver-operating characteristic (ROC) curves of age, BMI, hemoglobin, albumin, hs-CRP, and CEA (T0) for mortality prediction Age: AUC=0.515; p=0.843; cut-off value=39.5 years; sensitivity=100%; specificity=10.2%. BMI: AUC=0.579; p=0.313; cut-off value=21.42 kg/m^2^; sensitivity=73.3%; specificity=49.7%. Hemoglobin: AUC=0.719; p=0.005; cut-off value=125 g/L; sensitivity=66.7%; specificity=79%. Albumin: AUC=0.519; p=0.805; cut-off value=41.5 g/L; sensitivity=40%; specificity=73.2%. Hs-CRP: AUC=0.601; p=0.198; cut-off value=5.5 mg/L; sensitivity=46.7%; specificity=81.5%. CEA: AUC=0.805; p<0.001; cut-off value=34.61 ng/ml; sensitivity=60%; specificity=90.4%. AUC: area under the ROC curve; BMI: body mass index; Hs-CRP: high-sensitivity C-reactive protein; CEA: carcinoembryonic antigen

The ROC curve model in Figure 1 shows that the serum CEA level before surgery was a good factor to predict mortality in right colon cancer patients with TNM stages II and III (AUC=0.805, p<0.001, cut-off value=34.61 ng/ml, sensitivity=60%, and specificity=90.4%).

**Figure 2:**
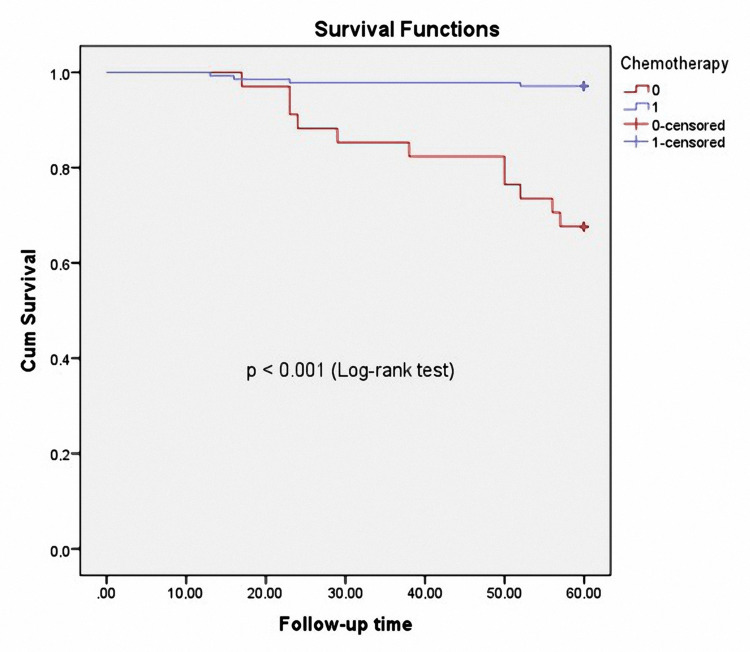
Kaplan-Meier analysis of the mortality of 172 patients, classified according to the groups with and without adjuvant chemotherapy (ACT)

Based on the Kaplan-Meier analysis in Figure 2, our results show that group 1 (red line) exhibited a significantly higher death rate compared to that with group 2 (blue line) (log-rank test, p<0.001).

## Discussion

Results of laparoscopic surgery and D3 lymph node dissection in right colon cancer patients with TNM stages II and III

CME and central vascular ligation of the feeding vessel, commonly known as D3 node dissection, are used by many surgeons to radically treat colon cancer, including elderly patients [5-7,12]. This technique can improve long-term survival and reduce local recurrence compared with previously performed procedures [8-10]. Currently, the technique is considered an extensive oncological surgery option for surgery for right colon cancer in many countries [12,17,20,21]. One of the most important prognostic factors of colorectal cancer is nodular status. This study has 49.4% of patients with lymph node metastasis (Table 1). A thorough dissection of D2 and D3 lymph nodes reduces the rate of metastasis and increases the patient's survival time [22].

This surgical method has few complications. Only 18 patients (10.46%) had early complications (data are not shown). Hwang et al. evaluated the results of laparoscopic complete mesocolic excision with D3 lymph node dissection for right colon cancer in 80 elderly patients (age ≥70 years) compared to those in 127 younger patients (age <70 years), and the overall complication rate in this study was 16.3% in the elderly group and 12.6% in the younger group [12].

Outcomes of laparoscopic surgery and D3 lymph node dissection during the five-year follow-up

Changes in Some Hematological and Biochemical Indices After One Year of Treatment

The results of our study showed that the percentage of patients with BMI <18.5 increased (26.2% vs. 16.9%), the anemia rate increased (42.4% vs. 32.6%), and the rate of albumin reduction <35.0 g/L increased (33.7% vs. 26.7%). However, the percentage of patients with elevated hs-CRP decreased (15.1% vs. 29.7%), and the rate of elevated CEA also decreased (46.5% vs. 55.8%) before and one year after the radical surgery (Table 1 and Table 2). CEA is a class of oncofetal antigens, produced within a normal fetus but only in trace amounts by normal adult cells [23]. In patients with adenocarcinoma, especially colorectal cancer, there is often an increased serum CEA level because more than 90% of primary colorectal cancers produce CEA [24]. Our results show that after one year of colon cancer resection, CEA levels and CEA elevation rates decreased together. This result is also consistent with those of Konishi et al. published previously [25].

Outcomes of Laparoscopic Surgery and D3 Lymph Node Dissection During the Five-Year Follow-Up

After the five-year follow-up, we had 11.6% of patients with recurrence, 10.5% of patients with metastasis, and a mortality rate of 8.7% (Table 2). Recurrence after the surgery of colon cancer was reported to be about 20-30% in 2008 and 2009 [26,27], but the recurrence rate after laparoscopic surgery in the study of Wang et al. in 2020 was only 7.1% (44/619 stage II colon cancer patients) during a median postoperative follow-up period of 42.2 months [28]. Our recurrence rate is higher than those in other studies. We think that our study group has both stage II and III colon cancer, and up to 34/172 patients (19.8%) refused ACT use after laparoscopic surgery. We counted the number of patients who died from all causes after five years of follow-up, and the results showed that 15/172 (8.7%) patients died (Table 2). Thus, our five-year survival rate is 91.3%. Our research results are similar to those of Hwang et al. conducted in 2020 (in terms of research subjects and surgical methods). In Hwang et al.'s study, the five-year survival rate of patients ≥70 years old was about 89% and that of the <70-year-old group was about 93% [12].

By understanding the predictors of mortality after surgery, we found that CEA is a factor with a good predictive value. CEA concentration before surgery had a predictive value for mortality (AUC was 0.805; p<0.001; Figure 1). Some previous studies also demonstrated CEA's role in predicting colon cancer patients' mortality after surgery [24,25]. CEA is a specific protein for gastrointestinal adenocarcinoma, including colon cancer. Although people without colon cancer still maintain a small amount of CEA in the blood, when the concentration is increased indirectly, there is an abnormal proliferation of colon cancer cells. In patients, the process of cancer development takes place silently, starting from the individual cell (this stage has not been detected on imaging methods, such as ultrasound, multi-energy CT, or positron emission tomography (PET) CT), only when the cancer cells gather to form a mass, the above imaging methods can detect it. Thus, it is possible to use serum CEA as a predictor of postoperative cancer recurrence or mortality in patients with right colon cancer stages II and III.

Role of ACT in reducing the ratio of recurrence and mortality

ACT after surgery for colorectal cancer is recommended by the NCCN and applied in most countries worldwide. In Vietnam, patients are advised to use ACT with the FOLFOX regimen after radical surgery for stages II and III of colon cancer. Although the patients were all explained of the effects of using ACT after surgery, there were still 34/172 (19.8%) patients who refused to use ACT after surgery. When comparing the clinical and laboratory characteristics before surgery of the group using and not using ACT, we found no difference in all indices, except for the proportion of patients with serum albumin <35.0 g/L (serum albumin levels in the group not using ACT were statistically significantly lower than the group that used it, with p=0.028, but the mean serum albumin concentration was not different, with p=0.073) (Table 1). The two groups had some laboratory index differences after one year of surgery. The group without ACT had a higher rate of anemia, increased CEA levels and reduced hemoglobin levels, and higher CEA concentrations than those with ACT (p<0.001). During the five-year follow-up, the recurrence, metastasis, and death rates in the group without ACT were also higher than those with ACT (p<0.001) (Table 2). In particular, when we used Kaplan-Meier analysis of mortality in the 172 patients, comparing the groups with and without ACT after surgery, we found that the ACT group had a higher rate of all-cause mortality lower than the group that did not use ACT (p<0.001) (Figure 2).

The role of ACT in reducing the recurrence rate and increasing the survival time of patients with colon cancer after surgery has been mentioned in many previous studies and in different countries worldwide [29,30]. Our study results confirm the role of ACT in reducing the recurrence rate and increasing the survival time for patients with stage II and III right colon cancer after laparoscopic surgery and D3 lymph node dissection.

Limitations

This is an observational study of treatment results in a hospital, so the number of patients is not much, especially because there is a difference in the number of patients between the ATC and non-ATC groups. The study has not distinctively mentioned the complications during and after surgery enough, so it has not comprehensively evaluated the patients' surgical results.

## Conclusions

From the results, we conclude that using laparoscopic surgery and D3 lymph node dissection combined with ACT reduced the five-year recurrence and mortality rates compared with the non-ACT group in patients with advanced-stage right colon cancer. The rates of recurrence, metastasis, and mortality in the non-ACT group after surgery were 41.2%, 38.2%, and 32.4%, respectively, higher than those in the ACT group (4.3%, 3.6%, and 2.9%, respectively; p<0.001). Preoperative serum CEA levels are a good predictor of five-year recurrence and mortality. After the radical surgery, ACT reduced recurrence and mortality in the patients with stage II and III right colon cancer.
